# Inequalities in glycemic management in people living with type 2 diabetes mellitus and severe mental illnesses: cohort study from the UK over 10 years

**DOI:** 10.1136/bmjdrc-2021-002118

**Published:** 2021-09-08

**Authors:** Jayati Das-Munshi, Peter Schofield, Mark Ashworth, Fiona Gaughran, Sally Hull, Khalida Ismail, John Robson, Robert Stewart, Rohini Mathur

**Affiliations:** 1 Department of Psychological Medicine, King’s College London, Institute of Psychiatry, Psychology and Neuroscience (IoPPN), London, UK; 2 South London and Maudsley (SLaM) NHS Trust, London, UK; 3 ESRC Centre for Society and Mental Health, King’s College London, London, UK; 4 School of Population Health & Environmental Sciences, King’s College London, Faculty of Life Sciences & Medicine (FOLSM), London, UK; 5 Department of Psychosis Studies, King’s College London, Institute of Psychiatry, Psychology and Neuroscience (IoPPN), London, UK; 6 Clinical Effectiveness Group, Institute of Population Health Sciences, Queen Mary, University of London, London, UK; 7 Department of Non-Communicable Disease Epidemiology, London School of Hygiene & Tropical Medicine, London, UK

**Keywords:** schizophrenia, diabetes mellitus, type 2, epidemiology, glycated hemoglobin A

## Abstract

**Introduction:**

Using data from a a primary care pay-for-performance scheme targeting quality indicators, the objective of this study was to assess if people living with type 2 diabetes mellitus (T2DM) and severe mental illnesses (SMI) experienced poorer glycemic management compared with people living with T2DM alone, and if observed differences varied by race/ethnicity, deprivation, gender, or exclusion from the scheme.

**Research design and methods:**

Primary care data from a cohort of 56 770 people with T2DM, including 2272 people with T2DM and SMI, from London (UK), diagnosed between January 17, 2008 and January 16, 2018, were used. Adjusted mean glycated hemoglobin (HbA1c) and HbA1c differences were assessed using multilevel regression models.

**Results:**

Compared with people with T2DM only, people with T2DM/SMI were more likely to be of an ethnic minority background, excluded from the pay-for-performance scheme and residing in more deprived areas. Across the sample, mean HbA1c was lower in those with T2DM and SMI (mean HbA1c: 58 mmol/mol; 95% CI 57 to 59), compared with people with T2DM only (mean HbA1c: 59 mmol/mol; 95% CI 59 to 60). However, HbA1c levels were greater in Bangladeshi, Indian, Pakistani, and Chinese people compared with the White British reference in the T2DM/SMI group. People with T2DM/SMI who had been excluded from the pay-for-performance scheme, had HbA1c levels which were +7 mmol/mol (95% CI 2 to 11) greater than those with T2DM/SMI not excluded. Irrespective of SMI status, increasing deprivation and male gender were associated with increased HbA1c levels.

**Conclusions:**

Despite a pay-for-performance scheme to improve quality standards, inequalities in glycemic management in people with T2DM and SMI persist in those excluded from the scheme and by gender, ethnicity, and area-level deprivation.

**Significance of this study**

**What is already known about this subject?**

- People with severe mental illnesses (SMI) experience major inequalities, dying 15–20 years earlier than the general population, mostly from preventable physical causes, with a higher prevalence of type 2 diabetes mellitus (T2DM) noted.
- There is some evidence that pay-for-performance schemes are associated with improvements in care.

**What are the new findings?**

- Using longitudinal data from a pay-for-performance scheme, we found that overall, glycemic management was improved in people living with T2DM and SMI compared with people living with T2DM only, however other inequalities persisted.
- In the group with T2DM and SMI, mean HbA1c levels were higher in some of the minority ethnic groups compared with the White British reference. In addition, those excluded from the scheme had markedly poorer glycemic management than those not excluded.

**How might these results change the focus of research or clinical practice?**

- Even in settings operating pay-for-performance schemes to incentivize high-quality care, clinicians should be aware that inequalities in T2DM care persist, and adversely impact people living with comorbid SMI.
- Outreach approaches (rather than exclusion) may be needed to improve diabetic care in people living with T2DM comorbid with SMI, in settings operating pay-for-performance schemes.

## Background

People with severe mental illnesses (SMI), such as schizophrenia spectrum and bipolar disorders, experience large reductions in life expectancy, mostly from preventable causes.^1 2^ The prevalence of type 2 diabetes mellitus (T2DM), obesity, hypertension, and metabolic syndrome is markedly elevated in these populations. This is due to a range of factors which may include the side effects of antipsychotic medications,^3^ as well as living with a debilitating condition which may make self-management of health conditions more challenging and adverse health behaviors more likely.^4^ The impact of deprivation^2 5^ and wider social determinants may also impact on health behaviors such as diet and physical exercise^3 6^ in these groups.

In our recent studies we have shown that relative to the general population, people living with SMI have a twofold to threefold elevated prevalence of T2DM, further elevated in Black Caribbean, Black African, Bangladeshi, Indian, and Pakistani people.^5^ In a recent study in the USA, diabetes prevalence was also noted to be higher in racial/ethnic minority groups with SMI compared with the White non-Hispanic American reference group.^7^ There is also a possibility that the presence of disparities due to SMI additively interacts with ethnic inequalities to worsen health outcomes.^5 8 9^ This may be further compounded by inequalities in accessing evidence-based treatments for T2DM management; for example, it has been shown that in the UK, South Asian and Black people with T2DM are less likely to receive indicated interventions, such as intensification of diabetic treatments, after persistently elevated glycated hemoglobin A1c (HbA1c) has been identified, compared with White British people.^10^ Previous work has also indicated worse glycemic management in people with T2DM in those of lower socioeconomic position^11–13^ and by race/ethnicity in the USA and other countries.^13–15^ In addition, although all care is free at the point of contact in the UK, one of the largest pay-for-performance schemes in the world (the Quality and Outcomes Framework (QOF)) operates, whereby family doctors/general practitioners are financially incentivized to deliver care on selected quality indicators in primary care.^16 17^ Although this scheme has led to some improvements in care, including reductions in health inequalities,^18^ emergency admissions,^19^ and potentially better monitoring of care in people with SMI and T2DM,^20^ concerns have been raised that family doctors/general practitioners may remove patients from pay-for-performance monitoring if the targets are felt by the clinician to be inappropriate to the patient; this practice is known as ‘exception reporting’.^16 17^ Concerns have been raised that exception reporting may be more likely in people with SMI as they may be more challenging to manage.^16 17 21^

There is a gap in the literature regarding potential inequalities impacting on glycemic management in people living T2DM and SMI, and most previous work has been cross-sectional^20^ or has been impacted through smaller sample sizes, limiting inferences.^22^ Therefore, using data from a large primary care cohort serving an ethnically diverse and geographically well-defined region in London, UK, with 10 years’ follow-up data, including repeated HbA1c assessments, we sought to assess disparities in glycemic management in people living with T2DM and SMI, taking into account specific inequalities relating to ethnicity, gender, area deprivation, and primary care exception reporting practices. We hypothesized that the presence of disparities across a range of indicators (presence of SMI, ethnicity, gender, deprivation, and mental health exception reporting) would be associated with poorer glycemic management, compared with reference groups.

## Methods

### Participants and setting

Primary care data from the London boroughs of City and Hackney, Tower Hamlets, and Newham were used for the present analyses. Primary care data are managed by primary care staff and include entries made by primary care doctors, nurses, and other clinicians. The boroughs are urban, inner-city areas in the top decile for deprivation in East London, with high ethnic diversity. In the UK, most people (approximately 98%) are registered with a general practitioner/family doctor. General practitioners coordinate physical and mental healthcare and take the lead in monitoring and managing T2DM, although they may also refer to diabetologists for additional advice. All communications related to care and changes to medications/prescribing are relayed back to the general practitioner. Since 2004 UK general practitioners have been incentivized through the QOF scheme to maintain a registry of people with T2DM. Introduction of this scheme has improved coding for conditions like T2DM because of QOF financial incentives.^23^ Using primary care Read codes as defined by QOF, a cohort of people with newly diagnosed T2DM were identified for the study. The cohort for the study was defined as any individual with an incident diagnosis of T2DM any time between January 17, 2008 and January 16, 2018, aged 40 years or older. We restricted to 10 years of glycemic management data (taking incident T2DM diagnosis as the ‘start’ point). This was undertaken to ensure higher data quality and also permitted an assessment of HbA1c management soon after diagnosis.

### Exposures

The main exposure for this study was presence of SMI. SMI diagnoses were identified in the primary care record using Read codes. In general, SMI diagnoses are made in secondary mental healthcare services by clinicians according to the International Classification of Mental Disorders-10 (ICD-10) criteria,^24^ with these diagnoses then passed back to primary care. SMI disorders in this study were defined as schizophrenia-spectrum disorders, bipolar disorders, or any non-organic psychoses, and mapped on to ICD-10 codes F2* (schizophrenia spectrum disorders), F30 (manic episode), F31 (bipolar affective disorder), and F32.3 (severe depressive episode with psychotic symptoms). Previous work examining the accuracy of identifying non-organic SMI diagnoses using electronic health records in primary care has indicated high levels of accuracy and completion.^25^

### Outcomes

HbA1c measurements are conducted and checked by clinicians in primary care at regular intervals. The frequency of measurements may range from 3-month to 6-month intervals until HbA1c levels and antidiabetic therapies are stable, at which point frequency of monitoring may change to 6 monthly.^26^ Repeated measurements of HbA1c (mmol/mol) over time were assessed in the cohort from the date of T2DM diagnosis to up to 10 years after diagnosis, when assessed at any time point in the study observation window. For the analyses, we used the date of each HbA1c assessment (which was the date that HbA1c was assessed and recorded in the clinical record by clinicians) to explicitly model HbA1c assessments in a repeated measures/multilevel modeling framework as described in the Statistical Methods section, which follows.

### Covariates

Demographic measures used in the study included age, gender, and deprivation. Area-level deprivation was defined according to quintiles of the Townsend deprivation score,^27^ with higher scores indicating greater area-level deprivation, assessed at lower super output area level, which has a mean of 1500 households.^28^ The Townsend score is an area-based socioeconomic deprivation measure derived from the UK national census data on residential and car ownership, unemployment, and overcrowding.^27^ It is grouped into national quintiles of deprivation. In this study we scored the first fifth as the least deprived and the last fifth as the most deprived. Ethnicity is recorded in primary care electronic records using self-reported ethnicity based on the UK Office for National Statistics criteria.^29^ This is collected at initial patient registration or during routine consultations using standard data entry templates,^29^ leading to the following groups: White British, Irish, Black African, Black Caribbean, Indian, Pakistani, Bangladeshi, and Chinese. ‘Mixed’ ethnicity groups were grouped according to the ethnic minority group indicated, consistent with approaches previously taken in national surveys of health of ethnic minority people in England.^30^ People indicating ‘other’ ethnicity were excluded from the sample due to the heterogeneity by ethnicity within this group (online supplemental figure 1). In addition, we also identified people who had been ‘exception-reported’ from mental health QOF indicators at any time during the observation window. Within this context, the practice of exception reporting is important because it is associated with the exclusion of patients from pay-for-performance schemes such as the QOF and may be used by practices to avoid financial penalties.^31^ Primary care physicians may use exception reporting for a range of reasons, including exclusion due to non-attendance after two appointments have been sent, patient refusal, if a secondary care service/investigation is unavailable, if a patient does not tolerate medications, or if there are other clinically inappropriate reasons to include them in monitoring/reporting.^31^ Prescriptions for antidiabetic medications are well captured in the primary care record, as general practitioners coordinate repeat prescriptions even if they are initiated in secondary care. Details on prescriptions were extracted from the primary care record, and a variable derived which indicated either any prescription of insulin (with or without oral hypoglycemic medications), any prescription of oral hypoglycemic medication (without insulin) and non-insulin injectable agents, or no antidiabetic medications (either oral or insulin), in the observation window. For each of the treatment approaches we assessed any mention of the treatment approach over the 10-year period. With intensification approaches (eg, no medication to non-insulin to insulin), the highest level was prioritized. Prescriptions for antipsychotic medications were also noted if dispensed within the study observation window period. The total number of consultations over the study observation period was noted. We also estimated ‘duration of T2DM diagnosis’ by subtracting the date of T2DM diagnosis onset from the end date for the study.

10.1136/bmjdrc-2021-002118.supp1 Supplementary data

### Statistical methods

To assess the association of the main exposure (SMI) with estimated mean HbA1c, while taking into account the correlation of repeated HbA1c measures in the same individual, mixed effects regression models with random intercepts and slopes were used, with hierarchical levels which accounted for repeated measures (level 1) nested in individuals (level 2), further nested in general practices (level 3). Random intercepts were specified at each level, and random slopes specified for repeated HbA1c measures in individuals over time. We compared models specifying random intercepts only with models specifying random intercepts and random slopes for repeated HbA1c measurements by individuals using likelihood ratio tests. Likelihood ratio tests supported the additional specification of random slopes to models; therefore, all models presented in this paper are mixed effects models with random slopes on repeated HbA1c measures in individuals. To assess whether the presence of any of the potential disparity indicators (ethnicity, gender, deprivation, and mental health exception reporting) modified the association of SMI with mean HbA1c estimates, we assessed the interactions for each of these indicators with the SMI variable. In fully adjusted models, there was strong evidence supporting a statistical interaction for SMI*ethnicity (Likelihood Ratio (LR) test χ^2^ (7)=16.67; p=0.02) and for SMI*exception reporting (LR χ^2^ (1)=6.62; p=0.01). Therefore, in final adjusted models we retained these two interactions and present stratum-specific estimates for these covariates. The *xtmixed* suite of commands in STATA-MP V.15 was used to build multilevel regression models adjusting for dates of HbA1c measures, age, gender, area-level deprivation, and with interactions (SMI*ethnicity and SMI*exception reporting), leading to adjusted estimates of mean HbA1c differences compared with reference groups. The *margins* command was then used to derive predicted mean HbA1c by ethnicity and exception reporting, stratified by the presence of SMI. We repeated the analyses within strata of T2DM treatments (ie, restricted to samples receiving no pharmacological treatment, oral hypoglycemic medication, or insulin). Finally, to assess whether receipt of antipsychotics, number of consultations, or duration of T2DM illness impacted on the estimates, we repeated the analyses adding these variables separately to the models. Across all models, likelihood ratio tests were used to assess the strength of associations and statistical interactions.

## Results

Data from 124 practices ranging from 808 to 15 650 registered people (mean 5395) were used for the analysis. In total 56 770 people with newly diagnosed T2DM between January 2008 and January 2018 were included in the study (online supplemental figure 1 details the composition of the final cohort used for analysis). The mean duration of T2DM in the cohort was 3174 days or 8.7 years. The mean duration of T2DM was similar in people with T2DM only (3174 days or 8.7 years) compared with people with T2DM/SMI (3176 days, 8.7 years). Of the cohort 4% (n=2272) had an SMI diagnosis.

**Figure 1:**
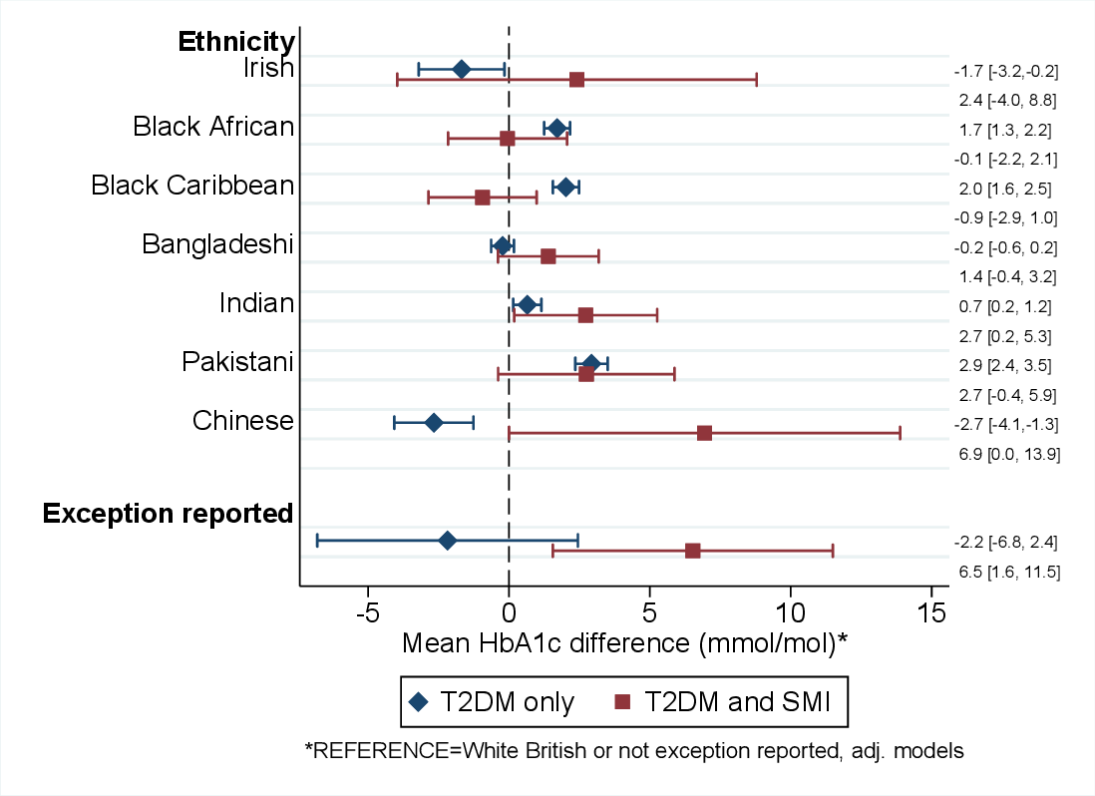
Mean HbA1c differences (mmol/mol) by ethnicity, exception reporting, and severe mental illness. Estimates adjusted for displayed variables (SMI*ethnicity and SMI*exception reporting interaction) and age, sex, date of HbA1c assessments, and area deprivation (continuous). See online supplemental table 1 for the models. HbA1c, glycated hemoglobin; SMI, severe mental illnesses; T2DM, type 2 diabetes mellitus.

Table 1 highlights the demographic characteristics of the sample. In general, in the cohort of people living with both SMI and T2DM, the mean age was lower, with more women and more people of Black Caribbean ethnicity in this group, than in the group with T2DM alone. The group with SMI and T2DM was over-represented in higher deprivation areas and had a greater proportion of people receiving insulin and more likely prescribed antipsychotics than those living with T2DM alone.

**Table 1:** Demographic characteristics of the sample

	Type 2 diabetes mellitus only		Type 2 diabetes mellitus with severe mental illness		
	n	%	n	%	
Total	54 498		2272		
Age (years), mean (SD)*	63	14	60	13	*F*=110.29, p<0.001
Gender					
Female	24 916	46	1146	50	Pearson’s χ^2^=19.58, p<0.001
Male	29 582	54	1126	50	
Ethnicity					
White British	12 363	23	578	25	Pearson’s χ^2^=184.08, p<0.001
Irish	399	1	24	1	
Black African	7440	14	326	14	
Black Caribbean	6710	12	455	20	
Bangladeshi	16 105	30	569	25	
Indian	6697	12	189	8	
Pakistani	4319	8	110	5	
Chinese	465	1	21	1	
Townsend deprivation score					
Least deprived, Q1	11 597	21	306	13	Pearson’s χ^2^=88.57, p<0.001
Q2	11 690	21	481	21	
Q3	10 616	19	500	22	
Q4	10 595	19	501	22	
Most deprived, Q5	10 000	18	484	21	
Exception-reported†					
Never	54 457	100	1958	86	Pearson’s χ^2^=6631.11, p<0.001
At least once	41	0	314	14	
Antidiabetes medications					
Diet controlled	7084	13	269	12	Pearson’s χ^2^=15.84, p<0.001
Oral/non-insulin injectable	38 529	71	1563	69	
Insulin	8885	16	440	19	
Prescribed antipsychotics					
None	53 245	98	608	27	Pearson’s χ^2^=2.25, p<0.001
Any	1253	2	1664	73	
Total consultations over the study period, mean (SD)	8.9	18.7	14.5	27.1	*F*=186.59, p<0.001
Number of HbA1c assessments over the study period, mean (SD)	9.9	7.1	10.6	7.4	*F*=18.36, p<0.001

*On date of extraction (January 2018).

†From mental health QOF indicators.

HbA1c, glycated hemoglobin; Q, quintile; QOF, Quality and Outcomes Framework.

Table 2 illustrates the estimated mean HbA1c in people with T2DM only versus T2DM/SMI, displayed for the full sample and then stratified by ethnicity and exception reporting, adjusting for all confounders. In the full sample with T2DM/SMI, the mean HbA1c was lower than in those living with T2DM only. By ethnicity, for the White British group with T2DM/SMI, the mean HbA1c was 57 mmol/mol (95% CI 55 to 58) and was lower than in the White British group with T2DM only (adjusted HbA1c: 59 mmol/mol; 95% CI 58 to 59), with similar trends observed in the Black Caribbean and Black African groups. Of note, in the group with T2DM/SMI who had been exception-reported/excluded from the pay-for-performance scheme, the mean HbA1c levels were markedly higher (62 mmol/mol; 95% CI 61 to 64) than those with T2DM/SMI who had not been exception-reported (58 mmol/mol; 95% CI 57 to 59) and also higher compared with those with T2DM only who had also been exception-reported (57 mmol/mol; 95% CI 52 to 62).

**Table 2:** Glycemic management in people with type 2 diabetes mellitus only and with severe mental illness, adjusted models

Estimated HbA1c (mmol/mol)	Type 2 diabetes mellitus only		Type 2 diabetes mellitus with severe mental illness	
	Mean	95% CI	Mean	95% CI
Full sample	59.2	58.9 to 59.5	57.9	57.2 to 58.6
Ethnicity				
White British	58.5	58.1 to 58.9	56.5	55.1 to 57.9
Irish	56.8	55.3 to 58.4	57.3	51.2 to 63.4
Black African	60.2	59.8 to 60.7	58.2	56.4 to 60.0
Black Caribbean	60.5	60.1 to 61.0	57.4	56.0 to 59.0
Bangladeshi	58.3	57.9 to 58.7	57.7	56.3 to 59.1
Indian	59.2	58.7 to 59.7	59.8	57.5 to 62.1
Pakistani	61.5	60.9 to 62.0	62.0	59.1 to 64.9
Chinese	55.9	54.5 to 57.3	61.0	54.3 to 67.7
Exception reported				
Not exception reported	59.2	58.9 to 59.5	58.0	57.1 to 59.0
Exception reported	57.0	52.3 to 61.6	62.4	60.6 to 64.2

Full sample estimates are from multilevel regression models, adjusted for age, sex, date of HbA1c assessments, and Townsend Deprivation Index. For stratified estimates HbA1c was estimated from multilevel regression models, adjusted for age, sex, date of HbA1c assessments, Townsend Deprivation Index, SMI*ethnicity, and SMI*exception reporting.

HbA1c, glycated hemoglobin; SMI, severe mental illnesses.

Across the cohort there were complex interactions with respect to the presence of T2DM/SMI and ethnicity. In people living with T2DM alone, relative to the White British reference group, Black Caribbean, Black African, Indian, and Pakistani ethnicity were associated with an increase in HbA1c levels, but were similar in the Bangladeshi group and lower in the Irish and Chinese groups (figure 1). In people living with both T2DM/SMI, compared with the White British reference group, adjusted HbA1c levels were elevated in Indian (+3 mmol/mol; 95% CI 0.2 to 5.2) and Chinese (+7 mmol/mol; 95% CI 0 to 14) groups (figure 1). HbA1c levels were also elevated in the Pakistani (+3 mmol/mol; 95% CI −0.5 to 6) and Bangladeshi (+1 mmol/mol; 95% CI −0.4 to 3.1) groups living with both T2DM/SMI compared with the White British reference; however, 95% CIs overlapped, indicating weaker evidence in support of a difference for these latter ethnic minority groups (figure 1). Of note, in the group with T2DM/SMI, exception reporting was associated with higher mean HbA1c levels (+7 mmol/mol; 95% CI 2 to 11) compared with those who had not been exception-reported.

**Figure 2:**
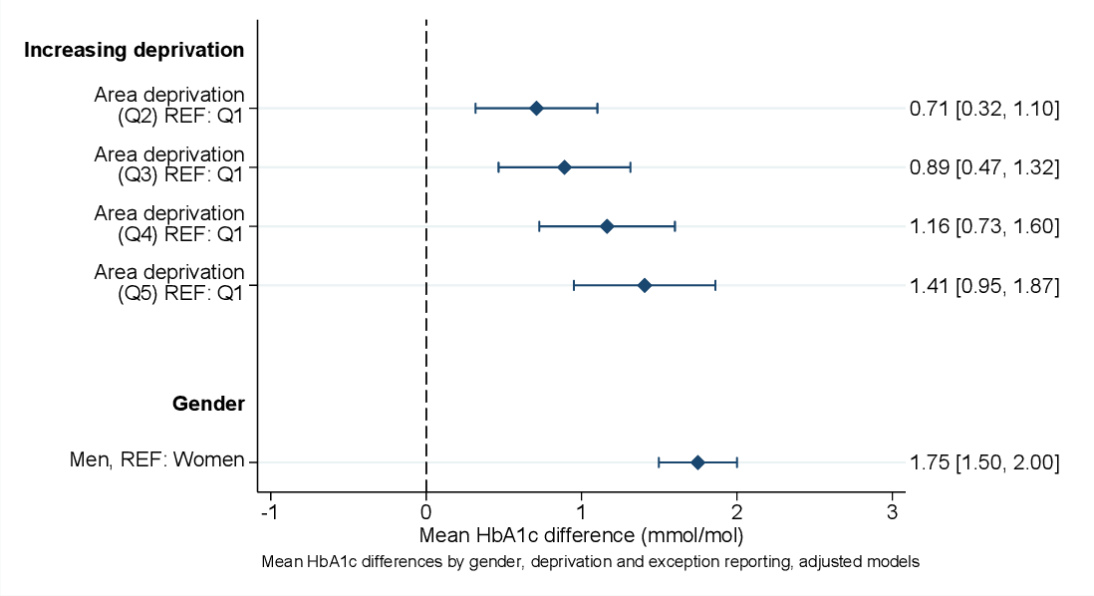
Mean HbA1c differences (mmol/mol) by gender and deprivation (full sample). Estimates adjusted for displayed variables (gender, deprivation in ordered categories), severe mental illness*ethnicity interaction, severe mental illness*exception interaction, age, and date of HbA1c assessments. See online supplemental table 1 for full estimates (crude/adjusted). HbA1c, glycated hemoglobin; Q, quintile; REF, reference.

Tests for statistical interaction did not indicate that the association of SMI with HbA1c levels was modified by either deprivation or gender. Therefore, these are displayed in figure 2 for the full sample, adjusted for all covariates including presence of SMI. Increasing deprivation was associated with a relative increase in HbA1c levels. Men compared with women had higher HbA1c levels (figure 2). The association of increasing deprivation with higher mean HbA1c followed a dose–response, with strong evidence in support of a linear trend (online supplemental table 1).

**Figure 3:**
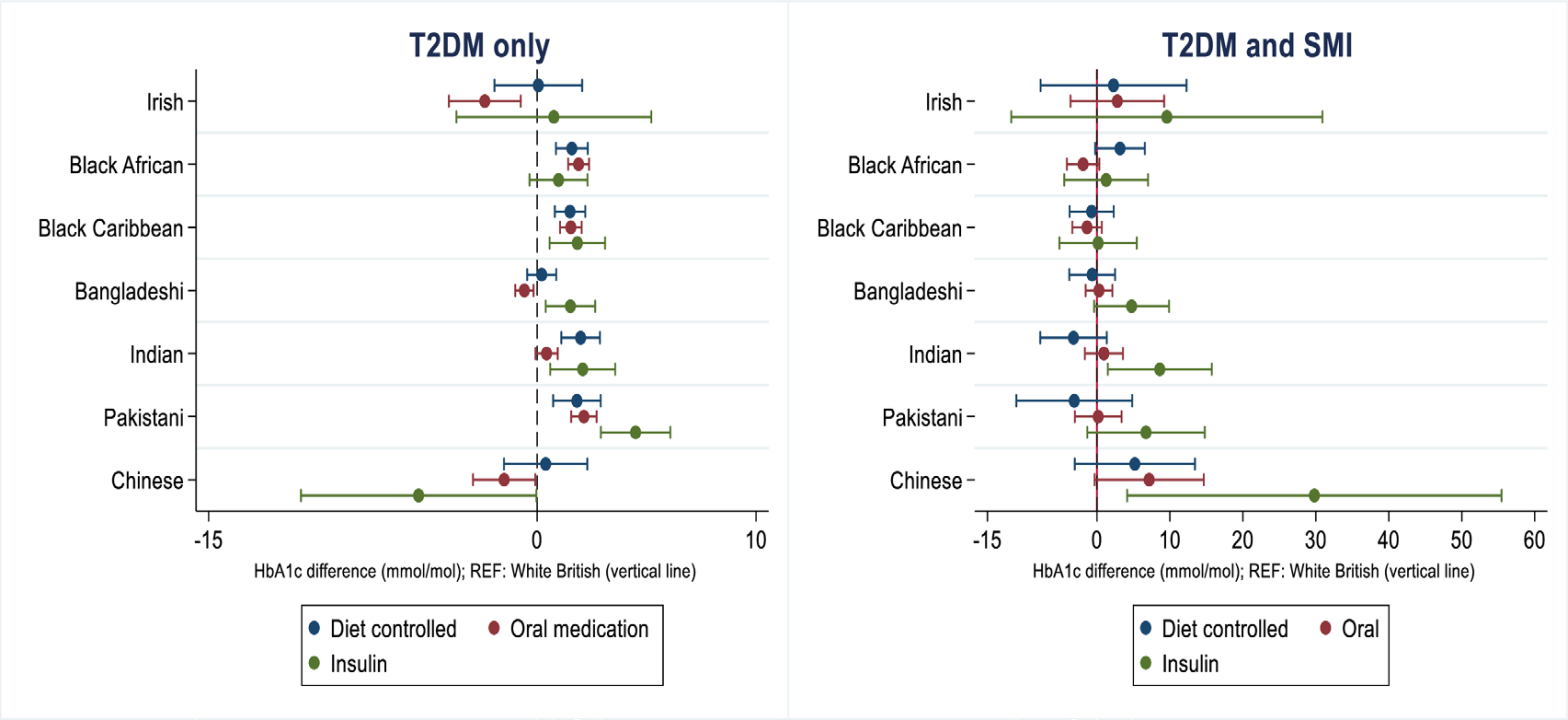
Mean HbA1c differences by ethnicity, severe mental illness and antidiabetic treatments. Adjusted for age, sex, exception reporting, Townsend Deprivation Index, date of HbA1c assessment, and antipsychotic medications. See online supplemental table 2 for estimates. HbA1c, glycated hemoglobin; REF, reference; SMI, severe mental illness; T2DM, type 2 diabetes mellitus.

Associations were assessed across the sample stratified by T2DM treatments, with an ethnicity*SMI interaction fitted (figure 3). In people on diet-controlled therapy, the ethnicity*SMI interaction was p=0.17, in people prescribed oral medication the ethnicity*SMI interaction was p=0.10, and finally in people prescribed insulin the ethnicity*SMI interaction was p=0.03, indicating strong evidence in support of an SMI*ethnicity interaction in the insulin-prescribed group. Figure 3 shows that in contrast to people living with T2DM only, in people living with both T2DM/SMI, glycemic management for some of the ethnic minority groups was close to parity with the White British group. However, this was not observed in Bangladeshi, Pakistani, Indian, and Chinese people with T2DM/SMI on insulin, who displayed higher HbA1c levels, relative to the White British reference group.

10.1136/bmjdrc-2021-002118.supp2 Supplementary data

Addition of ‘antipsychotic prescriptions’ or ‘total number of consultations over the observation window’ and ‘duration of T2DM’ variables to models had little effect on associations (see online supplemental figures 1 and 2), indicating that these variables did not account for observed associations. Finally, models were rerun without age restrictions (as people with SMI may develop T2DM at younger ages). These analyses did not impact on overall estimates (*available from authors on request*).

## Discussion

There are complex associations linked to glycemic management in people living with T2DM and SMI. First, our findings indicate that people living with T2DM comorbid with SMI do not necessarily experience poorer glycemic management compared with people living with T2DM only. This is consistent with previous findings from other studies in the UK^20^ and USA,^32 33^ and is unexpected given the observation that people with SMI die 15–20 years earlier than the general population, mostly from preventable physical causes.^1 2^ Unlike most previous work which has been cross-sectional,^20 32^ we were able to use longitudinal data with repeated measures of HbA1c levels, up to 10 years from diagnosis. Our study did not directly assess the before/after effect of introducing pay-for-performance approaches in UK primary care; however, it is possible that this has led to improved care for people living with T2DM comorbid with SMI. Previous work has indicated that the introduction of pay-for-performance schemes in primary care in the UK may have been associated with reductions in healthcare inequalities.^18 34^ However, the scheme has also been criticized, and concerns have also been raised that people living with multiple comorbidities are also more likely to be excluded from the scheme.^35^

A second set of findings related to the interactions with ethnicity in people with SMI and T2DM, which suggested differences across groups. In the group with T2DM/SMI, Bangladeshi, Indian, Pakistani, and Chinese ethnicity was associated with increased HbA1c levels, compared with the White British reference group. These differences in some instances were marked, ranging up to a large mean difference of +7 mmol/mol (95% CI 0 to 14) for the Chinese group with T2DM/SMI. In addition, we found that people living with T2DM/SMI who had been excluded from the pay-for-performance scheme through the practice of ‘exception reporting’ had HbA1c levels which were notably higher (+7 mmol/mol; 95% CI 2 to 11) compared with people living with T2DM/SMI who had not been exception-reported. This may indicate that outreach rather than ‘exception’ approaches are needed to effectively manage people with T2DM comorbid with SMI, although further work is needed to explore this. Irrespective of the presence of SMI, gender and residing in more deprived areas, were associated with higher HbA1c values. This is consistent with the wider literature^13^ and is noteworthy as people living with SMI in the cohort were also more likely to be residents of deprived areas. It should be noted, however, that while the differences relating to Chinese ethnicity and people who had been exception-reported were marked, many of the other differences in HbA1c management were more modest and less clinically significant.

We found that mean differences in HbA1c levels persisted and became more evident when analyses were further assessed according to diabetic treatments. Compared with people living with T2DM only, for some ethnic minority groups with T2DM/SMI, HbA1c levels were closer to that of the White British reference group across treatments. However, this was not noted in Bangladeshi, Indian, Pakistani, and Chinese people living with T2DM/SMI with a record of ever having received insulin, in whom HbA1c levels were noted to be higher compared with the White British group. Further work is needed to establish if there are ethnic disparities in T2DM intensification strategies,^10^ when also taking into account the presence of SMI, or whether these differences reflect earlier onset and more rapid/severe T2DM progression for some groups.

Observed differences by ethnicity may either indicate potential suboptimal management practices in the physical health of people with T2DM/SMI, which may include therapeutic inertia in intensifying diabetic treatments once high HbA1c levels are identified,^10^ and a higher risk of mortality or diabetic complications, potentially as result of ethnic inequalities impacting on the delivery of diabetes care.^36^ Alternatively, the findings may suggest more severe illness in these groups, which is then more challenging to manage. In a recent prospective study from the UK, the investigators noted that median HbA1c rose by 3.3 mmol/mol in Black and Asian patients in the first year after a psychosis diagnosis, with smaller increases of 1.1 mmol/mol in White patients.^37^ The investigators suggested a possibility of an ‘accelerated emergence of diabetes’ in people of an ethnic minority background, at younger ages, when diagnosed with psychotic disorders,^37^ an observation also supported by cross-sectional studies.^5^ Future work should aim to explore these important issues further, particularly as observed differences in suboptimal glycemic management, alongside other cardiovascular risk management targets (such as blood pressure, lipid profile, weight, tobacco cessation), are modifiable aspects of health management which could improve mortality outcomes in this group.^2 38 39^

The strengths of the study included its cohort design which enabled an assessment of glycemic management over time in people within the first 10 years of T2DM diagnosis. The large sample size of just under 60 000 people from primary care practices serving a large urban inner-city area in London, UK, ensured enhanced ethnic diversity, which enabled analyses that in previous work have not been possible. In the UK, although pay-for-performance schemes may incentivize the delivery of high-quality care to people living with targeted health conditions in primary care, overall healthcare remains free at the point of contact and most (98%) of the population are registered to primary care. As we used all data from a well-defined primary care catchment area, the study is therefore representative of the underlying population and we may be reasonably certain that selection biases will have been minimized, with the cohort reflecting the sociodemographic and ethnic composition of the population resident in the catchment of the study. The catchment area for the study was from one UK city; however, the setting is fairly typical and it is likely that findings could be generalizable to other metropolitan areas nationally. The inclusion of other potential indicators of social exclusion/marginalization, through assessment of the impact of exclusion from pay-for-performance schemes in primary care, enabled a broader assessment of inequalities impacting on glycemic management in people with T2DM/SMI, taking into account clinical practice. Further strengths of the study included our ability to assess whether the receipt of antipsychotic medications or the number of primary care consultations (as a measure of health system use/contacts) impacted on the associations. We found that although people with T2DM comorbid with SMI were more likely to be prescribed antipsychotic medications and had more consultations over the observation window compared with people living with T2DM alone, neither antipsychotic prescriptions nor consultations accounted for any of the observed associations for glycemic management.

There are however important limitations which need to be taken into consideration. Our study only assessed glycemic management in people with *known* T2DM under the care of general practitioners. Although primary care incentivization schemes ensure high levels of screening for T2DM,^40^ the finding of better glycemic management in the presence of SMI for some groups may be due to the possibility that our study only uses data from those in contact with healthcare providers who have been provided with monitoring. This is important as T2DM can be a ‘hidden’ condition and people with these diagnoses may not be in contact or known to healthcare services. Related to this, although QOF financial incentives have in general improved the coding for health conditions like T2DM in primary care, since their introduction in 2004,^23^ concerns have been raised about primary care coding practices impacting on the accuracy of identifying incident T2DM diagnoses.^23^ In a previous study, estimates for T2DM were found to be inflated when non-diagnosis codes were used to identify T2DM cases.^23^ In the present study we only used Read codes for T2DM diagnoses to identify and define the cohort; therefore, the risk of including people in the cohort who did not have T2DM would have been minimized. Compared with many previous studies in this area, our study was relatively well powered to assess ethnic inequalities; however, for some of the smaller ethnic minority groups (eg, Chinese people), our study was still limited, although suggesting the possibility of specific ethnic disparities which should be explored in future work. In addition, over the period of the study, although QOF was the most visible incentive scheme, there were multiple changes to national and local incentivization schemes which may have further impacted management and screening.^41–43^ These occurred against a backdrop of changes in national treatment guidance recommendations via the UK National Institute for Health and Care Excellence (NICE)^26^ and other programs such as the National Health Service Diabetes Prevention Programs (NHS DPP)^44^ and NHS Health Check,^45^ which may have also influenced management and screening. It would be beyond the scope of the current study and potentially impossible to disentangle these impacts, but these should be borne in mind when assessing the findings from the present study. The basis of our study on routine electronic health records data from primary care meant that we could not directly assess the impact of some indicators (eg, socioeconomic position assessed at the individual level) on outcomes. Although across all models we assessed associations while considering a range of confounders (eg, finding that antipsychotic prescriptions, number of consultations, and duration of T2DM did not confound estimates), it is possible that unmeasured confounding may still have biased estimates, for example body mass index and other somatic comorbidities, which were not assessed. We did not examine changes to treatment intensification approaches over time or severity of underlying T2DM, and so could not distinguish between these factors as potentially impacting on glycemic management. In addition, we only had access to data on mental health exception reporting; future work could also explore the impact of exception reporting for other conditions on health monitoring and management in people living with SMI and physical comorbidities. The present analyses focused on glycemic management; however, other indicators such as tobacco use, hypertension, weight gain, and hyperlipidemia play an important role in cardiovascular mortality risk^38^ and should be explored in future work for this group.

Future work should assess the impact of intersecting inequalities across multi-dimensions, for example the impact of deprivation and ethnicity, leading to inequalities in received care in people with T2DM comorbid with SMI. These are urgent concerns which, if directly tackled, could lead to improvements in earlier deaths in people with T2DM comorbid with SMI.

## Data Availability

Data may be obtained from a third party and are not publicly available. Data for this study are deidentified participant data. Data are not publicly available. Inquiries for data use should be directed to the authors and may be available subject to appropriate approvals.
