# Supplementary material for: Inequalities in glycemic management in people living with type 2 diabetes mellitus and severe mental illnesses: cohort study from the UK over 10 years
Source: BMJ Open Diabetes Res Care. 2021 Sep 8;9(1):e002118. doi: 10.1136/bmjdrc-2021-002118 (PMC8438718; doi:10.1136/bmjdrc-2021-002118)
Supplement: Supplementary data [file bmjdrc-2021-002118supp001.pdf]

SUPPLEMENTARY MATERIAL

Inequalities in glycaemic management in people living with type 2 diabetes mellitus and severe mental illnesses: Cohort study from the UK over ten years

Supplementary material: Tables 1; Figures 3

SUPPLEMENTARY FIGURE 1

Study flowchart

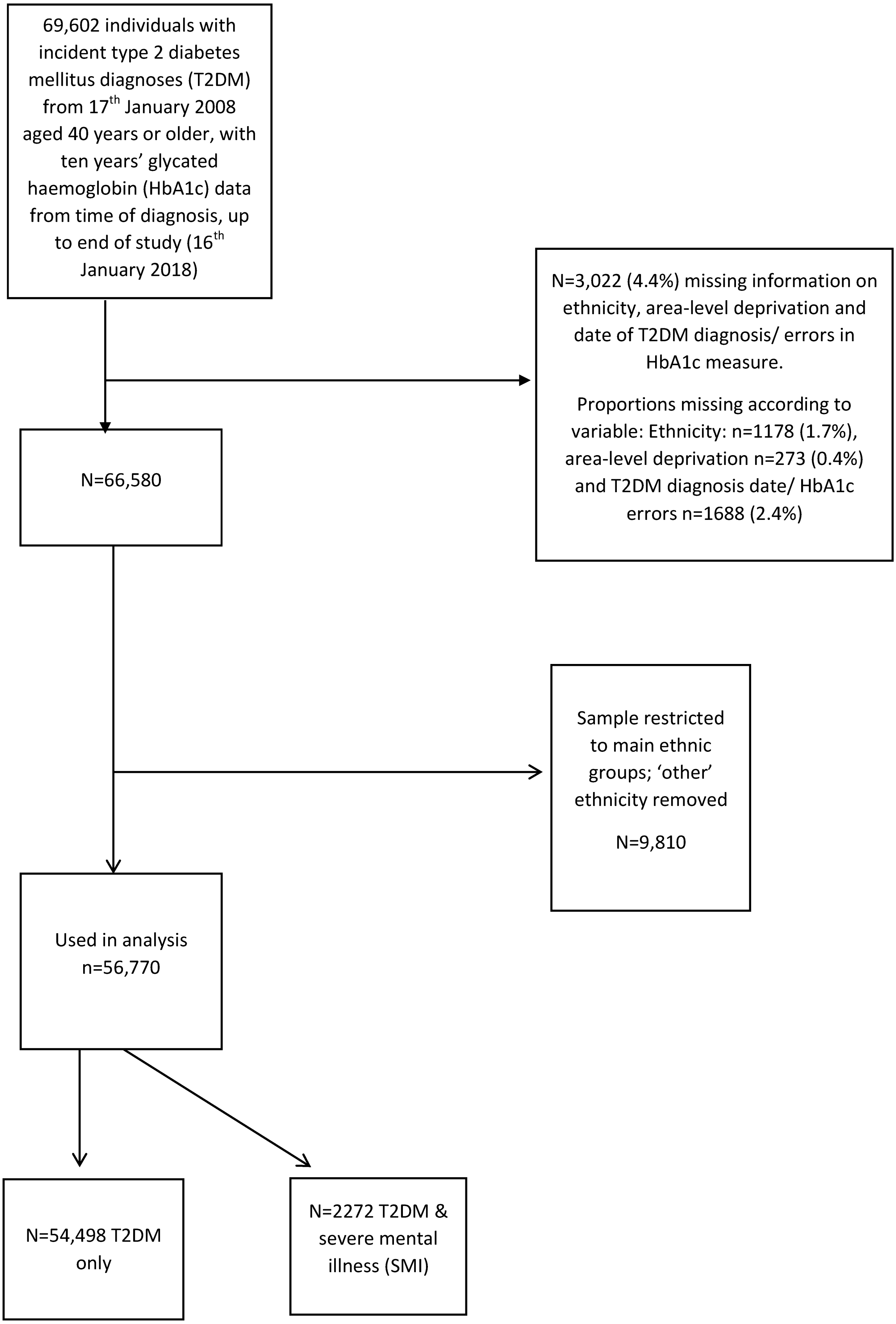

SUPPLEMENTARY Table 1: Crude and adjusted models; Association of demographic predictors with glycated haemoglobin (HbA1c) differences (mmol/ mol)

	CRUDE			ADJUSTED*		
	**b**	**(95% CI)**		**b**	**(95% CI)**	
**Ethnicity (T2DM only)**						
White British (REF)	REF			REF		
Irish	-2.02	-3.56	-0.47	-1.68	-3.21	-0.16
Black African	3.33	2.87	3.78	1.71	1.25	2.17
Black Caribbean	2.20	1.73	2.67	2.02	1.56	2.49
Bangladeshi	1.67	1.28	2.07	-0.23	-0.63	0.18
Indian	1.85	1.35	2.36	0.65	0.15	1.15
Pakistani	4.36	3.79	4.94	2.93	2.35	3.50
Chinese	-2.20	-3.62	-0.77	-2.67	-4.07	-1.26
**Ethnicity (T2DM & SMI)**						
White British SMI (REF)	REF			REF		
Irish SMI	2.29	-4.18	8.77	2.41	-3.97	8.79
Black African SMI	-0.73	-2.88	1.41	-0.05	-2.16	2.06
Black Caribbean SMI	-0.96	-2.91	0.99	-0.94	-2.86	0.98
Bangladeshi SMI	0.65	-1.16	2.47	1.40	-0.39	3.19
Indian SMI	2.04	-0.53	4.62	2.72	0.19	5.26
Pakistani SMI	1.75	-1.42	4.93	2.74	-0.39	5.87
Chinese SMI	7.55	0.51	14.58	6.94	0.00	13.88
**Age**						
Age (years)	-0.17	-0.18	-0.16	-0.17	-0.18	-0.16
**Gender**						
Male (vs. female)	2.06	1.81	2.32	1.75	1.49	2.00
**Exception reporting (T2DM only)**						
Not exception reported (REF)	REF			REF		
Exception	-3.30	-8.02	1.41	-2.18	-6.80	2.44
**Exception reporting (T2DM & SMI)**						
Not exception reported (REF)	REF			REF		
Exception	7.39	2.33	12.46	6.52	1.55	11.49
**Deprivation**						
Per quintile, increasing deprivation	0.33	0.22	0.44[t]	0.33	0.22	0.43[t]

Key: *P-value for association of all displayed variables was p<0.001 (including linear trend- indicated by t). *Adjusted models control for all displayed variables & date of HbA1c assessments and ethnicity*SMI and SMI*exception reporting interaction. Multi-level regression models adjusted for clustering by practice and repeated HbA1c measure. SMI: Severe mental illness; T2DM: Type 2 diabetes mellitus*

SUPPLEMENTARY FIGURE 2

Association of severe mental illness with glycated haemoglobin (HbA1c) measures, stratified by ethnicity and exception reporting. Models with and without adjustment for antipsychotic prescriptions, consultations and duration of T2DM diagnoses

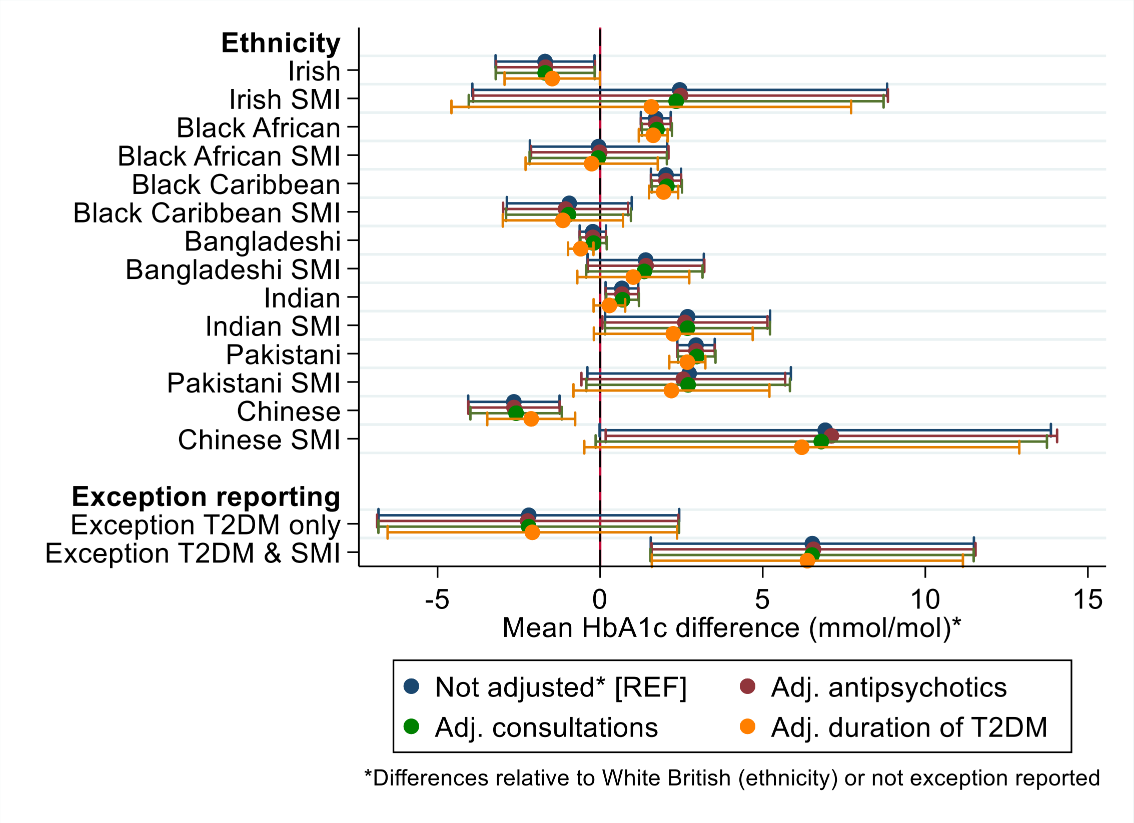

**All displayed estimates are adjusted for age, sex, deprivation, date of HbA1c assessments, ethnicity*SMI and exception reporting*SMI interactions. Red estimates display additional adjustments for use of antipsychotic medication, green estimates display additional adjustments for number of consultations over the observation period; orange estimates display additional adjustments for duration of T2DM diagnoses. T2DM: Type 2 diabetes mellitus; SMI: Severe mental illness.*

SUPPLEMENTARY FIGURE 3

Association of gender and area level deprivation with glycaemic management. Models with and without adjustment for antipsychotic prescriptions, consultations and duration of T2DM diagnoses

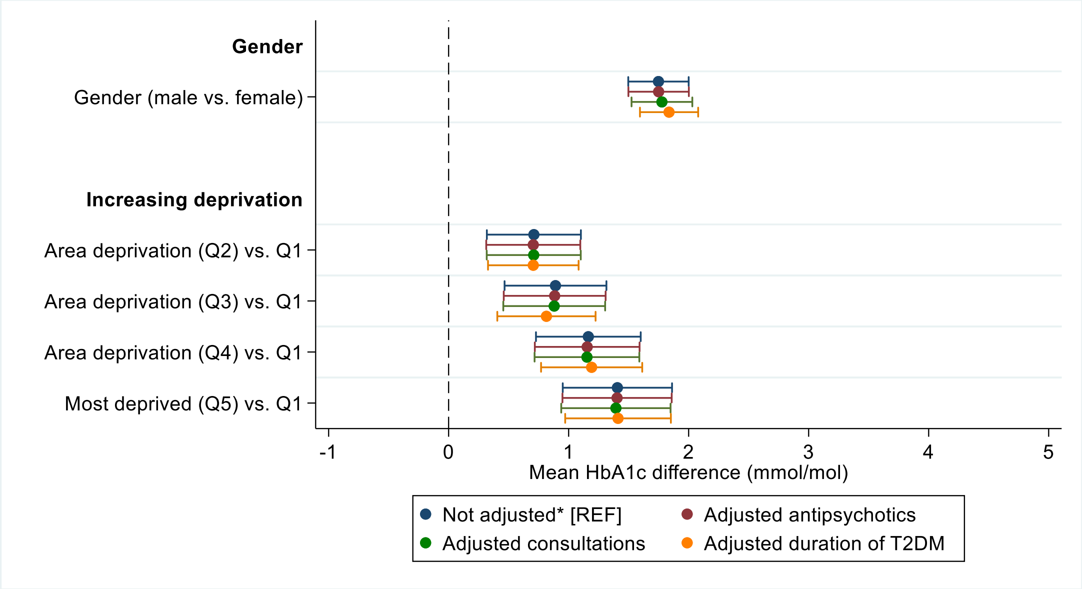

** All displayed estimates are adjusted for age, sex, deprivation, date of HbA1c assessments, ethnicity*SMI and exception reporting*SMI interactions. Red estimates display additional adjustments for use of antipsychotic medication, green estimates display additional adjustments for number of consultations over the observation period; orange estimates display additional adjustments for duration of T2DM diagnoses. T2DM: Type 2 diabetes mellitus; SMI: Severe mental illness.*
