# Supplementary material for: Inequalities in glycemic management in people living with type 2 diabetes mellitus and severe mental illnesses: cohort study from the UK over 10 years
Source: BMJ Open Diabetes Res Care. 2021 Sep 8;9(1):e002118. doi: 10.1136/bmjdrc-2021-002118 (PMC8438718; doi:10.1136/bmjdrc-2021-002118)
Supplement: Supplementary data [file bmjdrc-2021-002118supp002.pdf]

Supplementary table 2:

HbA1c differences by ethnicity (relative to White British reference) stratified by presence of severe mental illness and antidiabetic treatments

T2DM treatment type	T2DM/ SMI	Ethnicity	HbA1c difference (mmol/mol)*	(95%	CI)	p value
Diet controlled	T2DM only	White British	REF			
		Irish	0.06	-1.95	2.06	0.96
		Black African	1.59	0.86	2.31	<0.01
		Black Caribbean	1.50	0.81	2.2	<0.01
		Bangladeshi	0.21	-0.46	0.88	0.54
		Indian	1.99	1.11	2.87	<0.01
		Pakistani	1.82	0.73	2.9	<0.01
		Chinese	0.39	-1.51	2.29	0.69
	T2DM & SMI	White British	REF			
		Irish	2.27	-7.72	12.27	0.66
		Black African	3.16	-0.24	6.57	0.07
		Black Caribbean	-0.73	-3.75	2.3	0.64
		Bangladeshi	-0.65	-3.79	2.49	0.68
		Indian	-3.21	-7.77	1.35	0.17
		Pakistani	-3.11	-11.04	4.83	0.44
		Chinese	5.20	-3.03	13.43	0.22
Oral medication	T2DM only	White British	REF			
		Irish	-2.39	-4.03	-0.75	<0.01
		Black African	1.90	1.42	2.37	<0.01
		Black Caribbean	1.54	1.05	2.04	<0.01
		Bangladeshi	-0.58	-1	-0.16	<0.01
		Indian	0.43	-0.08	0.94	0.1
		Pakistani	2.14	1.55	2.72	<0.01

		Chinese	-1.50	-2.93	-0.08	0.04
	T2DM & SMI	White British	REF			
		Irish	2.79	-3.62	9.21	0.39
		Black African	-1.90	-4.12	0.32	0.09
		Black Caribbean	-1.35	-3.38	0.67	0.19
		Bangladeshi	0.28	-1.55	2.12	0.76
		Indian	0.95	-1.65	3.56	0.47
		Pakistani	0.17	-3.02	3.37	0.91
		Chinese	7.16	-0.33	14.65	0.06
Insulin	T2DM only	White British	REF			
		Irish	0.76	-3.69	5.21	0.74
		Black African	0.98	-0.34	2.3	0.15
		Black Caribbean	1.84	0.57	3.11	<0.01
		Bangladeshi	1.52	0.39	2.65	<0.01
		Indian	2.08	0.6	3.57	<0.01
		Pakistani	4.50	2.91	6.09	<0.01
		Chinese	-5.41	-10.79	-0.04	0.05
	T2DM & SMI	White British	REF			
		Irish	9.58	-11.74	30.9	0.38
		Black African	1.26	-4.48	7	0.67
		Black Caribbean	0.16	-5.14	5.46	0.95
		Bangladeshi	4.75	-0.38	9.88	0.07
		Indian	8.61	1.49	15.74	0.02
		Pakistani	6.72	-1.33	14.78	0.1
		Chinese	29.8	4.14	55.45	0.02

***Key:** Models have been adjusted for age, sex, exception reporting, Townsend deprivation index, date of HbA1c assessment and antipsychotic medications. SMI: Severe mental illness; T2DM: Type 2 diabetes mellitus; *Relative to White British reference*
